# PROTAX-GPU: a scalable probabilistic taxonomic classification system for DNA barcodes

**DOI:** 10.1098/rstb.2023.0124

**Published:** 2024-06-24

**Authors:** Roy Li, Sujeevan Ratnasingham, Iuliia Zarubiieva, Panu Somervuo, Graham W. Taylor

**Affiliations:** ^1^ Vector Institute for Artificial Intelligence, Toronto, Canada M5G 0C6; ^2^ Department of Computer Science, University of Toronto, Toronto, Canada M5S 2E4; ^3^ Centre for Biodiversity Genomics, Guelph, Canada N1G 2W1; ^4^ School of Engineering, University of Guelph, Guelph, Canada N1G 2W1; ^5^ Department of Biosciences, University of Helsinki, Helsinki 00014, Finland

**Keywords:** taxonomic classification, DNA barcoding, machine learning, high-performance computing

## Abstract

DNA-based identification is vital for classifying biological specimens, yet methods to quantify the uncertainty of sequence-based taxonomic assignments are scarce. Challenges arise from noisy reference databases, including mislabelled entries and missing taxa. PROTAX addresses these issues with a probabilistic approach to taxonomic classification, advancing on methods that rely solely on sequence similarity. It provides calibrated probabilistic assignments to a partially populated taxonomic hierarchy, accounting for taxa that lack references and incorrect taxonomic annotation. While effective on smaller scales, global application of PROTAX necessitates substantially larger reference libraries, a goal previously hindered by computational barriers. We introduce PROTAX-GPU, a scalable algorithm capable of leveraging the global Barcode of Life Data System (>14 million specimens) as a reference database. Using graphics processing units (GPU) to accelerate similarity and nearest-neighbour operations and the JAX library for Python integration, we achieve over a 1000 × speedup compared with the central processing unit (CPU)-based implementation without compromising PROTAX’s key benefits. PROTAX-GPU marks a significant stride towards real-time DNA barcoding, enabling quicker and more efficient species identification in environmental assessments. This capability opens up new avenues for real-time monitoring and analysis of biodiversity, advancing our ability to understand and respond to ecological dynamics.

This article is part of the theme issue ‘Towards a toolkit for global insect biodiversity monitoring’.

## 1. Introduction

Taxonomy, the science of classifying living organisms, has significantly evolved in recent years, influenced by major advancements in molecular biology and DNA sequencing technologies. This shift has led to a new era in which organisms are classified more on their genetic makeup than just on their physical traits. In this era, the merging of genomics with taxonomy has created the field of molecular taxonomy. This field aims to clarify the complex relationships among species, uncover unknown biodiversity, and improve our understanding of evolution. Access to these technologies is crucial to advancing our understanding of the planet. Estimates suggest that there are about 8.7 million (±1.3 million s.e.) species on Earth, with 86% on land and 91% in the oceans still undiscovered or unrecorded [1]. Developing and using automated taxonomic classification tools can help fill these knowledge gaps. These tools can accelerate the process of discovering, describing and cataloguing many unknown species, leading to a better understanding of global biodiversity.

### (a) Evolution of taxonomy and DNA barcoding

With around 3 billion (where 1 billion = 10^9^) specimens in global collections and 15 000–20 000 new species described each year, taxonomy qualifies as big data science [2]. Besides discovering new species, scientists are also finding new higher taxonomic lineages [3–5]. Occasionally, these new species do not fit neatly into existing taxonomic hierarchies, requiring a reevaluation of classification systems. However, the use of automated taxonomic methods and efficient computational and DNA sequencing tools has been limited, leading to a stagnant rate of new species naming. Furthermore, the limited resources in taxonomy make ongoing biodiversity cataloguing challenging. Species descriptions and morphological studies are not progressing as taxonomy experts age and retire [6]. Currently, this decline is happening faster than new resources are trained. Therefore, it is crucial to transition parts of the work to automated systems that can perform as well as or better than humans.

DNA barcoding is a method that automates species identification by using a short, standardized gene region to differentiate organisms. It relies upon variation among DNA sequences instead of physical characteristics, unlike traditional taxonomy based on morphology. This approach offers several advantages. It is more robust to phenotypic plasticity, is unaffected by sexual dimorphism, and is consistent across different life stages—all of which are challenges in morphology-based taxonomy. Owing to its effectiveness, DNA barcoding has become a key part of modern taxonomic identification systems [7,8]. For accurate species discrimination with DNA barcodes, there needs to be enough genetic variation between species. At the same time, variation within a species should be small to effectively delineate species. The mitochondrial gene cytochrome *c* oxidase I (COI) is suggested for this, as it closely follows this pattern [9]. For example, the average divergence between congeneric lepidopteran species was 6.8% [9]. However, variation within species is considerably lower, most likely owing to selective sweeps [10]. In the same study, the average COI divergence within lepidopteran species was just 0.25%. This feature makes COI a robust marker for species identification and explains its widespread use in DNA barcoding. DNA barcoding is becoming more important in inferring species interactions and in monitoring their response to environmental changes [9,11]. As a result, this technique has become essential in biodiversity research, leading to a rapid growth of DNA barcode records in public databases such as the Barcode of Life Data System (BOLD) and GenBank. For more information on BOLD see electronic supplementary material, §S1a.

### (b) Challenges in taxonomic classification

**Figure 1.**
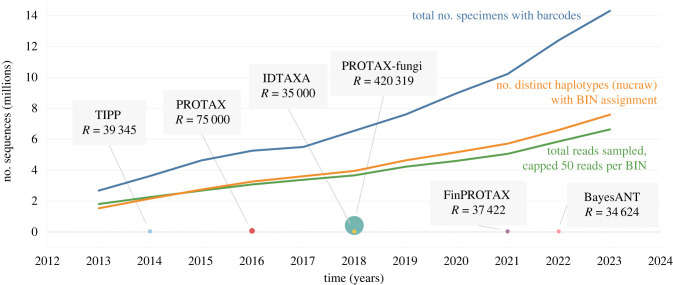
Display of the mismatch between BOLD’s data availability and the dataset size processed by various taxonomic classifiers: TIPP [12], PROTAX [13], IDTAXA [14], PROTAX-fungi [15], FinPROTAX [16] and BayesANT [17]. Here, *R* denotes the number of references (for non-parametric) or training sequences (for parametric) considered by a published algorithm. The reference dataset sizes in classifiers are depicted by the *y*-axis position and are proportional to the size of each corresponding circle. The total number of barcoded specimens in BOLD is shown by the blue line, with the count being influenced by significant oversampling in certain taxa. To facilitate a fairer comparison, the count of unique haplotypes is provided, also with read limits of 50 per BIN [18] imposed to prevent oversampling. Despite these practical measures, the available data volume significantly surpasses the reference datasets used by contemporary classifiers. (Online version in colour.)

The increasing use of DNA barcoding and advances in high-throughput sequencing have significantly increased the amount of sequence data available. As of September 2023, BOLD contains over 14 million barcode sequences representing over 1 million species. Analysing these constantly growing datasets is challenging both computationally and statistically, as larger databases often have more erroneous sequences and incorrect taxonomic information. Figure 1 illustrates the growth of the number of sequences in BOLD over the years and compares it with the data processed by various taxonomic classifiers in terms of their reference or training libraries. Despite BOLD’s increasing potential for growth and algorithmic innovation of taxonomic classifiers, newly proposed algorithms still develop and test on relatively small reference datasets.

Incomplete coverage in reference databases is a common problem when classifying organisms, particularly insects, where most species are unknown and many known species are absent [19]. Insects make up more than 70% of animal species and form the bulk of living organisms on Earth. As a result, traditional methods, like BOLD's Identification Engine, which match sequences to their closest matches, based on sequence identity, can make errors with insects and other less-studied groups.

Moving beyond traditional methods, several advanced algorithms have emerged, offering different approaches to the challenges of taxonomic classification. Taxonomic identification and phylogenetic profiling utilize statistical phylogenetic methods to identify sequences, adept at handling taxonomic novelty by integrating phylogenetic tree information [12]. IDTAXA applies *k*-mer-based similarity measures and a naive Bayesian classifier, excelling in microbial identification and demonstrating high accuracy even with novel taxa [14]. BayesANT (BAYESiAn Nonparametric Taxonomic classifier) combines Bayesian statistics with machine learning (ML) techniques, leveraging covariates such as sequence length and GC content to refine classification accuracy [17]. These methodologies represent the diverse strategies employed to navigate the complexities of large-scale genomic data and highlight the varying strengths and limitations each brings to the field of taxonomic classification.

Quantifying uncertainty appears to be an important strategy for correctly assigning sequences and interpreting environmental samples meaningfully. A clear example is the Malagasy insects of Madagascar, one of the world's highest biodiversity hotspots and home to a wide range of unique and endangered species [20]. However, the existing species classification system fails to account for much of the biological diversity there [21]. For example, even though Madagascar has a high concentration of ants, about 75% of its ant species are undocumented [22]. Therefore, classifiers used in such settings must not only handle previously unseen sequences but also accurately communicate the level of uncertainty in cases where the reference database is incomplete.

### (c) PROTAX and PROTAX-GPU

PROTAX [13] is a probabilistic model used for classifying DNA barcoding sequences. It has shown success in classifying arthropods in Finland, achieving 91% accuracy on 1000 unseen sequences [16]. PROTAX uses a Bayesian multinomial regression model that can incorporate any type of sequence similarity measure or outputs from other classifiers. It accounts for errors and gaps in the reference database, such as missing or unknown taxa and mislabelled sequences. This model offers a flexible probabilistic framework that blends classification with sequence similarity measures, converting them into probabilistic outputs. In the context of the challenges discussed previously, PROTAX’s probabilistic approach is particularly valuable. It not only allows the handling of new and uncharacterized sequences but also provides meaningful quantification of uncertainty when dealing with incomplete reference databases.

For COI sequences, PROTAX calculates pairwise distances between the query sequence and all reference sequences [16]. Being a semiparametric method that relies on nearest neighbours, PROTAX’s main limitation is its sequential process and dependency on pairwise comparisons, which makes it difficult to scale. To address scalability challenges, the wider ML community has made significant progress by using algorithmic parallelism and hardware accelerators. It is important to note that these innovations, especially the use of graphics processing units (GPUs), have been less integrated in bioinformatics, particularly in developing taxonomic classifiers. Our main contribution is to bridge this gap. We use scaling strategies from the ML and deep learning communities to enhance the PROTAX classifier, allowing it to efficiently handle larger reference databases with millions of records.

GPUs, essential for speeding up ML algorithms, offer powerful parallel processing capabilities that significantly accelerate computational tasks. While GPUs have transformed ML and artificial intelligence (AI) [23], their application in bioinformatics, particularly for DNA barcode analysis, remains underexplored. Our work demonstrates the effectiveness of GPUs in this domain, showing that PROTAX queries that previously took seconds or hours can now be processed in milliseconds.

This enables real-time identification of insects at a global scale, solving analysis bottlenecks in large-scale environmental sampling. Our approach’s higher throughput accelerates the discovery of new species, offers deeper insights into global biodiversity, and facilitates large-scale screening of environmental samples to find invasive species or assess ecosystem health. The ML community has shown that GPUs can greatly speed up tasks that are more compute-intensive than memory-intensive [24]. Since DNA barcode classification is similarly compute-bound, GPU-accelerated barcode classification offers a viable solution for scalable, real-time species identification.

This paper presents PROTAX-GPU, a parallelized enhancement of PROTAX using the JAX Python framework, achieving *ca*1000× faster processing of query sequences compared with the original. This advancement makes it possible to use BOLD-scale reference databases in DNA barcoding, while maintaining accuracy and memory efficiency.

## 2. Background

In this section, we review the PROTAX algorithm as introduced in [13]. We also detail its original central processing unit (CPU)-based implementation, which we refer to as PROTAX-CPU to distinguish it from our GPU-based implementation, PROTAX-GPU, described in §3.

### (a) PROTAX

**Figure 2.**
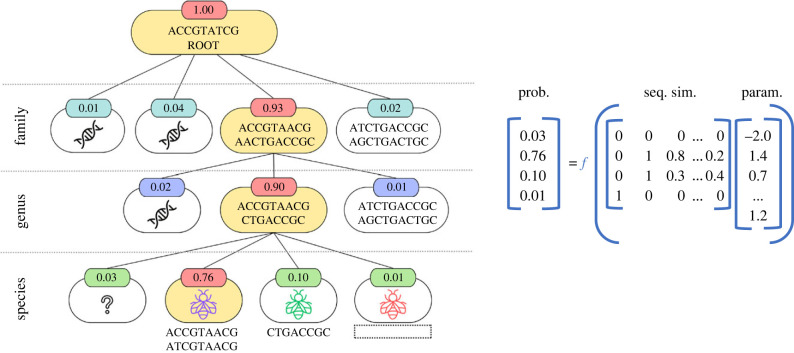
Overview of the PROTAX classifier. This model operates hierarchically from the root to the leaves of the tree, calculating probabilities at each non-leaf node based on predictors like sequence similarities or the outputs of other classifiers. (*a*) The hierarchical operation, with nodes in the path to the highest probability species highlighted in yellow. (*b*) The model for a genus-level node, illustrating how it allocates a probability (here, 0.9) across its branches. The design matrix displayed includes indicators for unknown taxa (0 0), known taxa with (0 1) and without (1 0) reference sequences, and sequence similarities. Softmax function *f* scales these probabilities, which are then integrated with the parent node probability. Parameter values, consistent within each taxonomic level, result from model training and remain fixed during query sequence classification. (Online version in colour.)

PROTAX is based on a hierarchical tree structure, where each node represents a different taxonomic unit (figure 2). It uses a reference sequence library, containing genetic sequences that correspond to these taxa. These sequences are linked to specific nodes, depending on their identified taxonomic level, which can range from species to broader categories like genus or family. PROTAX uniquely accommodates potential unknown taxa and the likelihood of mislabelled sequences in its library. This design enables PROTAX to manage uncertainties in taxonomic classification and address gaps or inaccuracies in reference libraries.

The PROTAX workflow begins by loading three types of input data: query sequence data, the relevant taxonomy, and a reference sequence database. The algorithm constructs a tree data structure from the taxonomy and operates hierarchically from the root to the leaves. This taxonomy outlines known species and their phylogenetic relationships, with leaves representing species and non-leaf nodes corresponding to broader taxonomic categories. At each node, PROTAX divides the probability among known branches and potential unknown branches, facilitating the estimation of probable outcomes based on the taxonomy.

The core of the PROTAX algorithm lies in its probabilistic model, which uses multinomial regression to calculate the likelihood of sequence placement within different taxonomic levels. It is parameterized by regression coefficients for each predictor, which can be either probabilities from an existing classifier or derived from sequence similarities between the query and descendant nodes at each branch. PROTAX can employ various strategies to utilize sequence similarities, such as nearest-neighbour similarity, average similarity of descendant nodes, or a combination of the two. Owing to the heterogeneity between different taxa, the use of average similarity may favour taxa with less diversity. However, using only the nearest neighbour may lead to misclassifications owing to mislabelled reference sequences. One possibility is to use the similarity from the nearest neighbour and the similarity from other neighbours, e.g. similarity representing a certain quantile. For instance, FinPROTAX [16] uses two predictors based on sequence similarity: one for the nearest neighbour and another for the difference between the nearest and the second-nearest neighbours. This approach of blending pairwise sequence similarities with a regression model is what categorizes PROTAX as a *semiparametric* method, which depends on both the taxonomy structure and learned parameters from reference sequences.

### (b) PROTAX-CPU

Most taxonomies, especially in biology, possess a complex hierarchical structure and dependencies. The dependencies reflect the evolutionary relationships, history and shared ancestry of taxa. Therefore, PROTAX was originally implemented as a sequential algorithm for the CPU, where its execution path naturally aligns with the hierarchical organization of taxonomic data [13].

In this paper, we evaluate two CPU-based implementations of PROTAX: PROTAX-CPU v. 1, and PROTAX-CPU v. 1.5 [25]. PROTAX-CPU v. 1 represents a baseline implementation of PROTAX that uses string representations of sequences in computing sequence distances. PROTAX-CPU v. 1.5 represents an optimized version of PROTAX that avoids recomputing overlapping sequence distances on children taxa via dynamic programming on all pairs of query and reference sequences, as well as using a binary representation of sequences for faster sequence distance computation (details in electronic supplementary material §S1b). Note that before computing sequence distances, following [13], the query and reference sequences are first translated into amino acid sequences, then aligned using a hidden Markov model (HMM), and finally back-translated into nucleotide sequences.

In both CPU implementations, the probability of each node in the taxonomy is computed in series, starting from the top of the tree, and working downwards. In addition, pairwise sequence distance computations between the query and every reference sequence are executed in a linear fashion by the original implementation, taking up the majority of the program runtime. This problem is exacerbated when considering the fact that BOLD is currently two orders of magnitude larger than the reference sequence database used in FinPROTAX [16]. The number of distance calculations scales linearly with the number of sequences as the reference sequence database grows. Thus, as datasets like BOLD grow in size, the computation taken by the original implementation of PROTAX increases proportionally to the number of entries it contains. Both versions of PROTAX-CPU already leverage approaches such as search space pruning. This allows PROTAX-CPU to skip nodes that do not meet a user-defined threshold, which can reduce its dependency on the size of the taxonomy. However, to enable analysis of modern large-scale biological datasets, transitioning from sequential to parallel computation is critical.

#### (i) Inference

To classify a query sequence **s**, PROTAX works in a hierarchical manner from the root of the taxonomy towards the leaves. At any given node *z*, PROTAX decomposes the probability assigned to *z* across the known branches and the possible unknown branches. The number of missing branches is unknown in reality, so we simply attach one unknown branch to each *z* as in [13].

Adopting the notation of [13], we denote the taxonomic levels of the nodes by *l* = 1, 2, …, *L*, where *l* = 1 is the coarsest level (e.g. class) and *l* = *L* is the finest level (e.g. species). Assuming node *z* at level *l* has *n*_*z*_ known branches, we split the unit probability into 1 + *n*_*z*_ parts: the first (*i* = 0) for the unknown branch and the rest (*i* = 1, 2, …, *n*_*z*_) for known branches. We use *Y*_*z*_ to denote the random variable indicating the branch to which sequence **s** belongs, with each *Y*_*z*_ modelled as a multinomial regression

2.1 $$P(Y_{z}=i)=\frac{w_{zi}\exp\left(\sum_{\;j=1}^{m}X_{ij}^{z}\beta_{j}^{z}\right)}{\sum_{i^{\prime }=0}^{n_{z}}w_{zi^{\prime }}\exp\left(\sum_{\;j=1}^{m}X_{i^{\prime }j}^{z}\beta_{j}^{z}\right)}.$$

Here, $\beta_{j}^{z}$ is the regression coefficient for predictor *j* = 1, …, *m* at node *z*. The (1 + *n*_*z*_) × *m* matrix **X**^*z*^, known as the design matrix, contains *m* predictors for classification. Weights *w*_*zi*_ can incorporate prior knowledge on missing branches, indicating parts of the taxonomy likely to be more complete. Following [16], we set *w*_*zi*_ to 1. As noted in §2a, any classification-informative predictors can be used. Adopting the nearest-neighbour predictor configuration of [16], we set each row of the design matrix to $X_{i}^{z}=\left[\begin{array}{c} b_{i1}^{z},b_{i2}^{z},r_{i1}^{z},r_{i2}^{z} \end{array}\right]$, where $r_{i1}^{z}$ and $r_{i2}^{z}$ are the sequence distances to the two closest reference sequences from the query within the descendants of branch *i*, with $r_{i1}^{z}$ being the closest, and *r*_*i*2_ the second-closest. If a node has only one reference sequence assigned to it, $r_{i2}^{z}$ simply takes the default value of 1. $b_{i1}^{z}$ and $b_{i2}^{z}$ are binary variables that indicate whether node *i* represents known or unknown taxa and whether or not it contains reference sequences. For all *i* = 1, …, *n*_*z*_, $b_{i1}^{z}=1$ if the *i*th node is known. In the case where node *i* represents an unknown taxon, $X_{i}^{z}=[0,0,0,0]$. In the case where node *i* has a reference sequence, $X_{i}^{z}=[0,1,r_{i1}^{z},r_{i2}^{z}]$.

Each predictor *j* is assigned a regression coefficient $\beta_{j}^{z}$. While in general the values of the regression coefficients $\beta_{j}^{z}$ can be node-specific, we follow the simplifying assumption in [13] that they are constant within each level and denote them by ***β***^*l*^. In our predictor configuration, this results in learning only four parameters per level: $\beta^{l}=[\beta_{1}^{l},\;\beta_{2}^{l},\;\beta_{3}^{l},\;\beta_{4}^{l}]$.

PROTAX allows for the potential misclassification of reference sequences. We use *I*(**s**) to denote the true species of sequence **s** and *I*′(**s**) for the assigned species identity (which may be incorrect). For any species identity *I*, *Y*(*I*) represents the outcome corresponding to the species identity, i.e. the complete taxonomic identification. Given a query sequence **s**, the probability that the observed outcome *Y*(*I*′(**s**)) equals outcome *y* is

2.2 $$P(Y(I^{\prime }(s))=y|\beta ,q)=qP(Y(I^{\prime }(s))=y)+(1-q)P(Y(I^{\prime }(s))=y|\beta ,I^{\prime }(s)=I(s)).$$

Here, *q* is the probability that a reference sequence is misidentified and *P*(*Y* = *y*) is the prior probability of the outcome *y*. The term *P*(*Y*(*I*′(**s**)) = *y*|***β***, *I*′(**s**)) is given by the product of the multinomial probabilities (equation (2.1)) from the root to the outcome *y*.

#### (ii) Parameter estimation

In PROTAX-CPU, Markov chain Monte Carlo (MCMC) sampling, specifically the Metropolis–Hastings algorithm, is used to obtain a *maximum a posteriori* (MAP) point estimate of the regression parameters, ***β*** and mislabelling probability *q*. Both ***β*** and *q* have an uninformative prior, $N(0,100^{2})$. Here, we also make the same assumptions and simplifications as in the original PROTAX paper [13] while parameterizing the model. In particular, the assumption that the mislabelling probability *q* = 0 at inference time. While MCMC scales for smaller PROTAX models, it struggles with larger taxonomies and more training data. Noting that parameter uncertainty was never exploited by the inference algorithm, PROTAX-GPU employs an alternative GD-based parameter learning strategy that is more suited for GPU execution.

## 3. Methods

In response to the limitations of PROTAX-CPU, we developed PROTAX-GPU using JAX, a high-performance Python machine-learning library optimized for hardware accelerators like GPUs and tensor processing units (TPUs). This implementation harnesses the parallel processing power of GPUs, enabling significantly faster sequence analysis while maintaining the flexibility and user-friendly interface of Python. PROTAX-GPU retains the core functionalities of PROTAX, including the integration of various classifiers and accuracy of taxonomic classification, but with enhanced computational efficiency and speed.

For detailed descriptions of JAX’s features, such as automatic differentiation, composable function transformations, and the integration of PROTAX-GPU with other Python tools, refer to electronic supplementary material, §S2a.

### (a) PROTAX-GPU

The general workflow of PROTAX-GPU remains largely consistent with that of PROTAX-CPU, though with a few algorithmic changes to leverage GPU acceleration. As in PROTAX-CPU, the inputs to the model consist of a query sequence to be compared against a taxonomy and a reference sequence database, where each reference sequence is assigned to a node and its ancestors. Here we provide a brief overview of the PROTAX-GPU workflow, while electronic supplementary material, §S2b describes each of these steps in detail.

**Figure 3.**
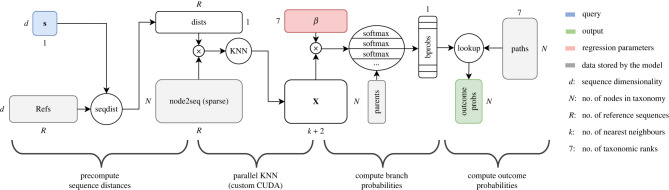
Data flow of PROTAX-GPU with respect to a query sequence, **s**. Symbols around each box specify matrix dimensions. The number of taxonomic levels in this case is specified as 7. The process can be conceptually subdivided into four stages. The sequence distance between a query sequence and all reference sequences is computed in parallel, with the *k* nearest neighbours (KNN) loaded into a design matrix. This design matrix is multiplied with regression parameters *β* before being converted to conditional branch probabilities, and combined into a product representing the joint probability distribution over assignments at every level. CUDA, Compute Unified Device Architecture. (Online version in colour.)

The computational pipeline for PROTAX-GPU is shown in figure 3. It begins with a comparison between a single query sequence, **s**, and each sequence in the reference database (details in electronic supplementary material, §S2b(i)). Next, these distances are assigned to their respective taxa via a single column-wise multiplication with a sparse binary matrix, which encodes all associations between taxonomic units (nodes) and reference sequences. This sparse matrix is then used as the basis for a parallel *k*-nearest-neighbours-like computation, which, for every node, yields the two closest sequence distances to the query over all child taxa, stored in the final two columns of the design matrix **X** (electronic supplementary material, §S2b(ii)). We finally perform a matrix-vector multiplication with the regression parameters, ***β*** over each taxonomic rank, to produce a vector of weighted sums for all taxa. The softmax function is then applied over each ‘neighbourhood’ of parent taxa and their children in order to obtain a probability distribution of ‘branch probabilities’ (electronic supplementary material, §S2b(iii)), which models the probability of the query being assigned a node given that it was assigned to its parent. Finally, we perform a lookup with a matrix that contains the indices of all branch probabilities in the path, starting from the root, to the branch probability corresponding to one row in the matrix. This corresponds to the ‘decomposition’ step in PROTAX, where we compute a product of the relevant branch probabilities according to the chain rule of probability (electronic supplementary material, §A2b(iv)).

### (b) Gradient descent

PROTAX operates on the foundation of a Bayesian multinomial regression model. Appropriately, PROTAX-CPU employs MCMC sampling with adaptive proposal distributions. Yet, past studies have used the MAP parameter estimate for classification, which does not exploit the modelling of parameter uncertainty. Moreover, as we show in our experiments, MCMC does not scale well to large reference libraries.

We therefore investigate training PROTAX-GPU via gradient descent (GD) as a scalable means of obtaining a point estimate of ***β***, leveraging the faster inference runtime provided by PROTAX-GPU, and the automatic differentiation capabilities in JAX. Specifics of the training process, including batch size, learning rate, and training data composition can be found in electronic supplementary material, §S2c.

### (c) Model evaluation

In our model evaluation, we assessed PROTAX-GPU using both real-world and synthetic datasets, focusing on species-level classification. We evaluated the model’s performance based on critical metrics like runtime, memory usage, and accuracy. The evaluation process involved running each reference sequence through both PROTAX-GPU and PROTAX-CPU to compare their classification accuracy at different taxonomic levels and to assess the predictive uncertainty as measured by perplexity. Further details on the datasets used, specific performance metrics, and the methodology for calculation are available in electronic supplementary material, §S2d.

## 4. Results

We evaluated PROTAX-GPU’s viability for automated taxonomic classification by comparing its accuracy and perplexity with two CPU implementations on the FinPROTAX dataset. Additionally, we assessed scalability by training it on BOLD and extending inference to a larger synthetic reference library of 13 million sequences. In every case, the CPU and GPU models have the same number of parameters, 4 × *L* = 28.

### (a) Comparison of PROTAX-GPU and PROTAX-CPU on the FinPROTAX dataset

Table 1 reveals that PROTAX-GPU is able to match and slightly exceed the performance of PROTAX in terms of accuracy and perplexity. The accuracy and perplexity values reported by PROTAX-GPU exactly match those of PROTAX, up to the family level. Additionally, PROTAX-GPU is able to achieve an accuracy of 97.4% at the species level, compared with PROTAX-CPU’s accuracy of 97.2%. This slight increase in accuracy seen in PROTAX-GPU is also reflected in the path accuracy in electronic supplementary material, table S1, in which PROTAX-GPU scores 99.5% compared with 99.48%. PROTAX-GPU-GD-37k, which was trained via GD, was able to achieve similar levels of accuracy to PROTAX-CPU-37k v. 1.5 and PROTAX-GPU-37k, with a species-level accuracy of 97.4%.

**Table 1.** Performance comparison of PROTAX-GPU and PROTAX-CPU on FinPROTAX dataset (37 422 aligned COI sequences). Accuracy and perplexity are computed over different taxonomic ranks with pre-trained regression parameters. Here, PROTAX-CPU v. 1.5 represents the optimized CPU implementation, and PROTAX-GPU represents the GPU implementation, using the same parameters. PROTAX-GPU-GD represents PROTAX-GPU inference with parameters learned by gradient descent (see §3b). Where there was a statistically significant difference, values in bold denote the best performing methods under the respective metric.

rank ...	1 (class)	2 (order)	3 (family)	4 (subfamily)	5 (tribe)	6 (genus)	7 (species)
**PROTAX-CPU-37k v. 1.5**							
accuracy (top 1)	1	1	1	0.9985	0.9981	0.9935	0.9721
perplexity	7.0	7.0156	7.0792	10.285	45.137	9.5989 × 10^4^	1.3348 × 10^35^
**PROTAX-GPU-37k**							
accuracy (top 1)	1	1	1	0.9985	0.9980	0.9935	**0.9735**
perplexity	7.0	7.0156	7.0792	10.285	45.137	9.5989 × 10^4^	1.3348 × 10^35^
**PROTAX-GPU-GD-37k**							
accuracy (top 1)	1	1	1	0.9984	0.9928	0.9922	**0.9739**
perplexity	7.0	7.0156	7.0792	10.285	45.137	9.5989 × 10^4^	1.3348 × 10^35^

To further substantiate the performance of PROTAX-GPU, we conducted an in-depth functional equivalence experiment comparing it with PROTAX-CPU. The results, including a detailed analysis of design matrices and path accuracy analysis, are presented in electronic supplementary material, §S3.

### (b) Training PROTAX-GPU on the BOLD dataset

As we scale up our reference sequences to the 7.8 million sequences from BOLD, we see that the performance of our *k* nearest neighbours (KNN) baseline model declines steeply after the family level, achieving only 34% species-level accuracy (table 2). This nearest-neighbour model simply assigns the query sequence to the taxon that contains the closest reference sequence in terms of sequence distance. While this decision rule may work for smaller, highly curated reference sequence databases with a low mislabelling probability, it clearly fails to perform well on more fine-grained classifications at the genus and species level for datasets such as BOLD, which is both larger and expected to be noisier than the FinPROTAX dataset.

**Table 2.** Comparison of evaluation metrics on the BOLD dataset (7.8 million aligned COI sequences) over each taxonomic rank in terms of accuracy. Here, PROTAX-GPU-GD-6M represents a PROTAX-GPU model trained on all available training data, while PROTAX-GPU-GD-3M represents a model trained on half of the training set. KNN, *k* nearest neighbours. Where there was a statistically significant difference, value in bold denotes the best performing method under the respective metric.

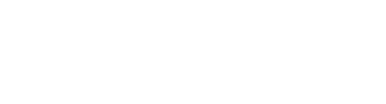	1 (phylum)	2 (class)	3 (order)	4 (family)	5 (subfamily)	6 (genus)	7 (species)
KNN-baseline-6M	1	1	0.97	0.92	0.88	0.80	0.34
PROTAX-GPU-GD-3M	1	1	1	0.9924	0.9883	0.9544	**0.9273**
PROTAX-GPU-GD-6M	1	1	1	0.9919	0.9878	0.9552	0.9245

By contrast, both GD-trained models have a significant accuracy over all taxonomic ranks, achieving a species-level accuracy of approximately 92%. While this is slightly lower than the species-level accuracy seen in the FinPROTAX dataset, this is to be expected for a larger, noisier taxonomy spanning a greater range of taxa. In addition, the performance of the GD-trained model does not degrade as rapidly when classifying at lower ranks, exhibiting only a 3% drop from the genus to species level, compared with the 50% drop in KNN-baseline-6M. It is worth noting that PROTAX-GPU-GD-3M, while trained on only half of all reference sequences in the training set and using the same taxonomy, is able to achieve a similar level of accuracy to the model with access to the entire training set (PROTAX-GPU-GD-6M).

GD was observed to be much more memory and runtime efficient in training PROTAX compared with MCMC, for both the small FinPROTAX dataset and the 13 million reference sequence dataset. For reference, computing the gradient of a minibatch over 37 422 reference sequences took 1.43 s, compared with 1 min for one iteration of MCMC. On top of this, for just the FinPROTAX dataset, 13.6 GB of VRAM was required in order to run MCMC, compared with the 1.1 GB used by GD. The difference in memory usage becomes more apparent as we scale to the larger BOLD dataset. Here, GD-based training only requires 5.6 GB of VRAM, while MCMC runs out of memory (see §3b).

### (c) Scaling up PROTAX-GPU to 13 million synthetic dataset representative of BOLD

**Figure 4.**
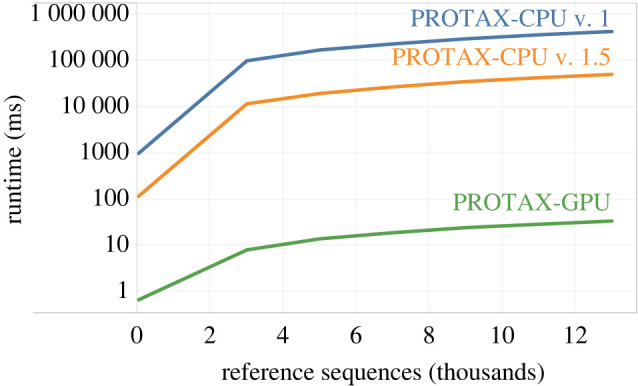
Runtime of single-query performance of PROTAX-GPU compared against two versions of PROTAX-CPU as a synthetic reference database scales to 13 million sequences. Runtime for each dataset is averaged over 10 runs. (Online version in colour.)

In terms of runtime performance, as illustrated in figure 4, we observe that PROTAX-GPU outperforms the PROTAX-CPU v. 1 and PROTAX-CPU v. 1.5 implementations when the number of reference sequences grows. In fact, sequence distance computations begin to dominate the runtime of PROTAX-GPU, taking up to 30 ms at 13 million sequences, while the time taken by KNN takes 5 ms. It is worth noting that the runtime of KNN appears as a piecewise linear function that changes slope initially but scales linearly for all reference sizes we tested. This may be owing to how JAX internally handles computational resources. While all implementations of PROTAX scale linearly with the reference sequence size (figure 4), it is evident that PROTAX-GPU grows at a much more relaxed rate compared with its CPU counterparts, fully classifying a query sequence against a reference sequence database of 13 million sequences in under 40 ms. In comparison, it takes the baseline CPU implementation of PROTAX 7 min to classify one query against 13 million synthetic sequences, and approximately 50 s for the optimized CPU implementation. It is the pruning of low-probability nodes that begins to dominate the runtime of the CPU-based implementations as the taxonomy grows. Pruning is unnecessary for the parallelized implementation, which allows PROTAX-GPU to achieve a more than 1000 × speedup compared with the most efficient CPU implementation, PROTAX-CPU v. 1.5, at 13 million reference sequences.

## 5. Discussion

Our study marks a significant advancement in scaling up taxonomic classifiers to meet the current size of reference and training libraries. This achievement bridges the critical gap between the vast amount of available data and the methods required to effectively interpret them, addressing a long-standing challenge in biodiversity research. By implementing PROTAX-GPU, we have enabled the processing of large-scale DNA barcode datasets, such as those in BOLD, with an unprecedented level of efficiency.

This progress in computational capability is key to tackling the complexities of taxonomic classification in the modern era of genomics. Our results show that PROTAX-GPU achieves a level of accuracy consistent with the original PROTAX while offering significantly faster processing speed and efficient memory use. This improvement enables quicker, real-time DNA barcoding, which is particularly beneficial for large-scale projects like BOLD. Importantly, PROTAX-GPU requires only 7.8 GB of VRAM to manage a taxonomy with 13 million reference sequences, fitting well within the capacity of most modern GPUs. This efficiency makes high-speed, scalable DNA barcoding more accessible, even on standard computer hardware.

PROTAX-GPU maintains the core functionality of PROTAX-CPU, including the ability to provide a probability distribution for each taxonomic classification. This feature is valuable as it offers insights into the confidence level of each identification made by the algorithm. While our evaluation primarily focused on the most likely identification, PROTAX-GPU’s strength lies in its capacity to present a range of possible classifications with corresponding confidence levels. This allows users to set thresholds for accepting identifications based on confidence, thereby minimizing errors. The ability to understand and convey the degree of uncertainty in identifications is particularly valuable in biodiversity studies, where precise species identification is often crucial.

A key advancement of PROTAX-GPU is its effective use of GD for training, as demonstrated in table 2. A question that naturally arises is: how much of the performance gains are owing to the parallelism and how much are owing to using GD? GD impacts training and parallelism impacts both inference and training. An end user on a system such as BOLD will only experience inference times when submitting queries. However, training typically requires much more computational resources than inference, and this becomes a concern for system maintainers especially when libraries are constantly updated, as is the case with BOLD. The runtime presented in figure 4 is with respect to inference. Therefore, the speedup observed there as well as the query times reported in §4c are owing to the parallelization of the algorithm. The runtime results reported at the end of §4b concern training. The speedup observed there is owing to the switch from MCMC to GD and the fact that GD is able to exploit GPU-based parallelism. Specifically, parallelization allows the probability distribution over the full tree to be computed quickly, which is needed to compute the loss in GD training. Therefore, the implementation of the parallel algorithm is significant as it not only accelerates inference but makes stochastic gradient descent (SGD) feasible.

The potential for integrating PROTAX-GPU into diverse genomic pipelines is promising. PROTAX’s adaptability has already been demonstrated with non-coding markers, notably in PROTAX-fungi, which utilizes the internal transcribed spacer (ITS) marker and represents the largest implementation of PROTAX prior to our work [15]. The ongoing collaboration with the PROTAX-fungi team to incorporate PROTAX-GPU into their workflows underscores this adaptability. This integration is expected to significantly enhance the handling of large-scale ITS marker datasets.

Porting of the HMM-based alignment pipeline to GPU accelerators as demonstrated recently by WFA-GPU [26] could further extend the applicability of PROTAX-GPU. This advancement would not only streamline the alignment process for various markers but also complement the GPU-accelerated classification capabilities of PROTAX-GPU. Such developments will make PROTAX-GPU a more versatile tool, capable of addressing the growing demands for rapid and accurate taxonomic classification in various fields of biological research.

## 6. Conclusion

In this study, we implemented PROTAX in JAX to create PROTAX-GPU, leveraging GPU acceleration to significantly enhance the speed of DNA barcoding analysis. Our implementation achieved over a 1000 × speedup compared with the optimized CPU version, fitting within the 7.8 GB of VRAM of a standard consumer-grade GPU.

This advance in computational efficiency has significant implications for environmental DNA (eDNA) analysis, which often involves processing large volumes of sequence data [27]. This tool enhances our ability to identify elusive or threatened species, detect invasive species, and improve biodiversity assessments with minimal habitat disturbance. By capturing a broader range of species diversity [28], eDNA analysis, complemented by efficient tools like PROTAX-GPU, becomes an indispensable asset in ecological research and conservation efforts.

We are committed to making PROTAX-GPU accessible and user-friendly. For the bioinformatics community, our open-source release on GitHub offers the flexibility to integrate PROTAX-GPU or its components into diverse projects, thanks to the Python-based framework. We aim to support the integration process through comprehensive documentation and community engagement.

For ecologists and conservationists, we are collaborating with the BOLD team to deploy PROTAX-GPU as a web-accessible tool. This effort will eventually allow PROTAX-GPU to replace BOLD’s current Identification Engine, providing users with a powerful yet user-friendly platform for DNA barcoding. Our goal is to ensure that developers and non-developers can efficiently use the enhanced capabilities of PROTAX-GPU in their work.

In conclusion, while PROTAX-GPU inherits the accuracy and reliability of PROTAX, its implementation marks a significant step towards addressing the growing demand for fast and efficient taxonomic classification. By making this tool accessible to a broad range of users, we aim to empower researchers and practitioners to make informed decisions based on reliable species identification, even when faced with large and incomplete datasets.

## Data Availability

Data and code is available from the GitHub repository: https://github.com/uoguelph-mlrg/PROTAX-GPU [29]. Supplementary material is available online [30].
